# Gel-aided sample preparation (GASP)—A simplified method for gel-assisted proteomic sample generation from protein extracts and intact cells

**DOI:** 10.1002/pmic.201400436

**Published:** 2015-02-05

**Authors:** Roman Fischer, Benedikt M Kessler

**Affiliations:** TDI Mass Spectrometry Laboratory, Target Discovery Institute, Nuffield Department of Medicine, University of OxfordOxford, UK

**Keywords:** Bottom-up proteomics, Comparative proteomics, Gel-assisted sample preparation, Sample preparation, Technology

## Abstract

We describe a “gel-assisted” proteomic sample preparation method for MS analysis. Solubilized protein extracts or intact cells are copolymerized with acrylamide, facilitating denaturation, reduction, quantitative cysteine alkylation, and matrix formation. Gel-aided sample preparation has been optimized to be highly flexible, scalable, and to allow reproducible sample generation from 50 cells to milligrams of protein extracts. This methodology is fast, sensitive, easy-to-use on a wide range of sample types, and accessible to nonspecialists.

Bottom-up or “shotgun” proteomics has a profound impact on modern biology and biomedical research, providing molecular windows underlying biological processes [1]. Such experiments essentially rely on the proteolytic digestion of biological material (e.g., protein extracts) as MS/MS-based sequencing of peptides remain the primary analytical approach using MS.

The two major sample preparation strategies include in-solution [2] and in-gel protocols [3,4], and they have recently been extended by filter-based [5,6] and pipette tip based methods [7]. These yielded increased sensitivity over conventional methods and allow compatibility with strong denaturants, such as SDS to maximize protein solubilization, in particular of membrane and chromatin-associated proteins. In-gel sample preparation is employed after the gel electrophoresis based separation of proteins and is widely used even outside laboratories specialized on proteome analysis. In-solution protocols are technically more challenging and usually involve chaotropic reagents, such as urea, to increase the solubilization of proteins, and are applied to analyze the “deep proteome” in shotgun/bottom-up proteomics [8].

In-gel sample preparation has been applied to copolymerized protein extracts in glass capillaries (Tube-Gel) by Lu and Zhu [4] to solubilize hydrophobic proteins from membrane preparations, leading to the development of a variety of “gel-assisted” [9–14] methods in which electrophoretic separation in a polyacrylamide matrix is omitted and the function of the polymer matrix is diminished to the effective containment and retention of protein material for washing and digestion steps. Gel-assisted approaches have been applied predominantly to homogenized and lysed tissue samples (brain [11], breast [12], or colon [13]) in the search for biomarkers focusing on membrane proteins and in combination with label-free and iTRAQ quantitation to exploit the solubilization of transmembrane, raft, and exosomal [14] proteins by gel-assisted methods.

We developed a gel-assisted method that was optimized and simplified compared to previous protocols [4,9–14], in which the complete and fast reaction of cysteine residues with monomeric acrylamide to form cys-S-β-propionamide (PAM-cys) [15] replaces the alkylation of cysteine residues with reagents, such as iodoacetamide or 2-chloroacetamide, that require additional processing steps. The proposed method limits the contact with the sample to a minimum to avoid contamination; minimizes sample loss; and is robust, scalable, sensitive, and easy to use for nonspecialists. We reasoned that the resulting method would have similar advantages as compared to “filter-aided” protocols, such as compatibility with high concentrations of detergents (i.e., SDS) and highly effective proteolysis or increased sensitivity over conventional in-solution methods. Besides the different mechanism for protein retention (filter vs. gel matrix) as demonstrated by Lu and Zhu [4], the working principle and workflow is very similar to “filter-aided sample preparation” (FASP), hence the use of an analogue acronym “gel-aided sample preparation” (GASP). However, FASP has limitations in total protein input, ease of use (i.e., repeated long centrifugation steps), or robustness (i.e., filter clogging or failure). We propose that gel-assisted methods do not suffer from these limitations and showcase GASP as an alternative to filter-assisted and in-solution methods, generating proteomic samples for MS analysis of equal or higher quality.

The essential elements of GASP are as follows: (i) protein extraction in presence of reducing agent, such as DTT, (ii) copolymerization of proteins with monomeric acrylamide, (iii) shredding of gel plug to increase surface area, (iv) depletion of small molecules, such as detergents and inhibitors, (v) proteolysis, and (vi) peptide recovery (Fig.1A). LC-MS/MS analysis was performed using a linear ion trap-orbitrap instrument (Orbitrap Velos, Thermo Scientific) [15] or a hybrid quadrupole-orbitrap instrument (Q Exactive, Thermo Scientific) [16] (details available in the Supporting Information Methods section). For the cutting of the gel plug into smaller pieces, we recommend a pulse centrifugation of the gel through a plastic grid, which can be obtained by removing the filter membrane from SpinX (Corning) filtering devices fitting into 1.5 mL tubes (i.e., by dissolving a cellulose acetate membrane in acetone) or using the also available version without filter membrane. This step is necessary to increase the effective surface area of the gel matrix for buffer exchange. The principle has been described by Lazarev et al. [17] using a hand-made device. For data analysis, we used different search algorithms (PEAKS, ProteinPilot, and MASCOT) to address specific experimental questions (for details see Supporting Information Methods).

**Figure 1:**
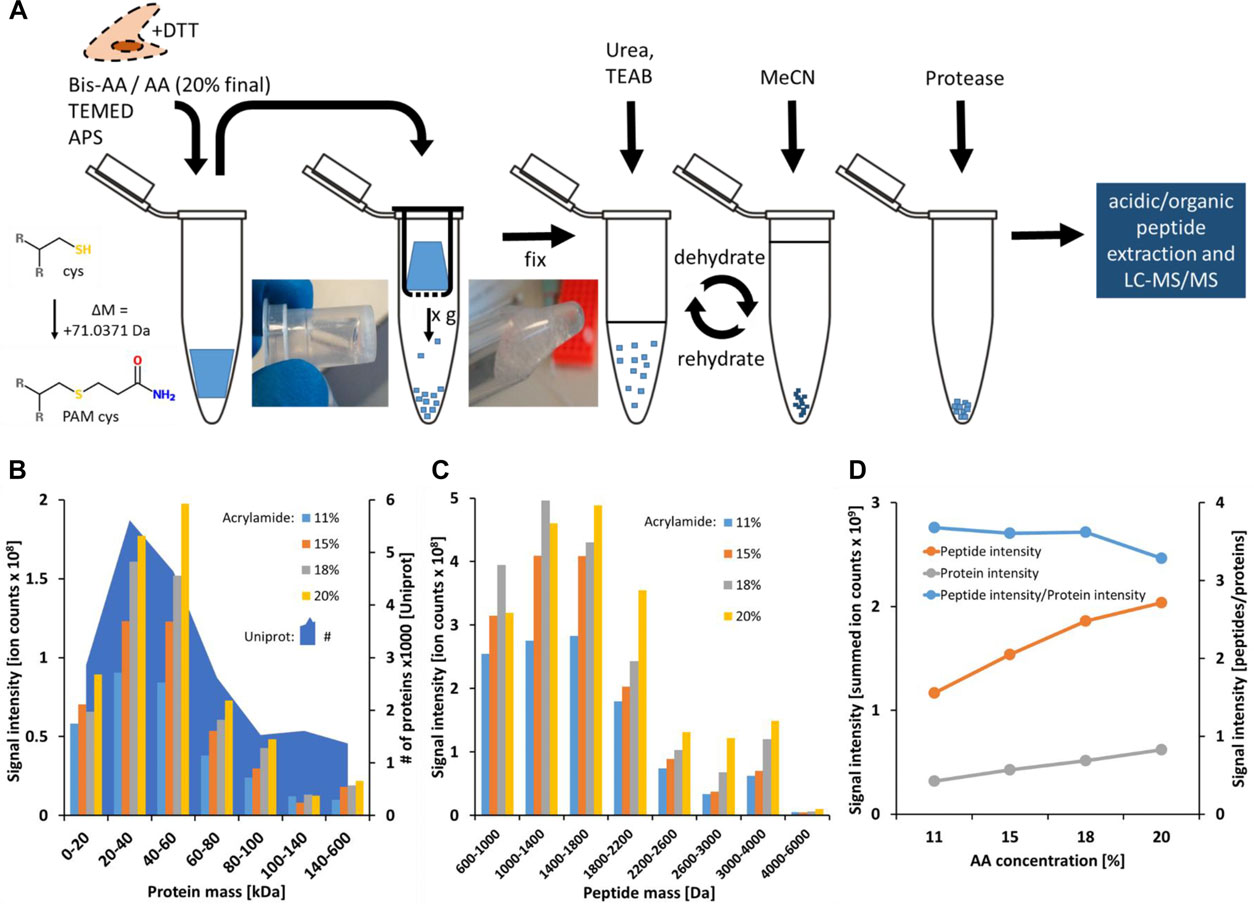
Gel-aided sample preparation (GASP) workflow. (A) Cell or tissue extracts are prepared in the presence of DTT to reduce disulphide bridges. Acrylamide/bis-acrylamide solution is added to a final concentration of 20%, by which cysteine residues are converted quantitatively to PAM-cys (propionamide). Polymerization is initiated by adding TEMED and ammonium persulfate. The gel block is then cut into small pieces by centrifugation through a plastic mesh to remove detergents and chaotropic reagents by further processing as in regular in-gel protocols (for details see Supporting Information Methods). GASP can be easily adapted to process high protein amounts, cell extracts, or intact cells. (B) Comparison of GASP at different acrylamide concentrations. The summed ion counts of identified, binned proteins show increased protein immobilization at higher acrylamide concentrations. The profile of identified proteins is similar to the mass distribution in the human proteome (secondary axis). (C) The summed ion counts of identified, binned peptides show better peptide recovery at higher acrylamide concentrations, due to the better immobilization of proteins. (D) GASP needs to negotiate protein immobilization and peptide recovery. At an acrylamide concentration higher than 18%, peptide recovery plateaus, while protein immobilization is further improved at 20%. As a compromise, we chose an acrylamide concentration of 20% for all further experiments.

In previous publications [4,9–14], acrylamide concentrations of ≤10% were used to immobilize proteins in a gel matrix, suggesting that peptide recovery may be the rate-limiting step for a sensitive detection of proteins rather than protein immobilization. To further optimize “gel-assisted” protein sample preparation, we evaluated the impact of the acrylamide concentration on the contrarian objectives “protein immobilization” and “peptide recovery” (Fig.1B to D).

The pore size of the polyacrylamide matrix varies depending on the concentration of bis-acrylamide [18]. To analyze whether the pore size has a selective effect on the molecular weight of the immobilized proteins, we compared the molecular weight of proteins identified by MS/MS after conducting GASP, using different bis-acrylamide concentrations (Fig.1B). As readout, we used the summed peptide ion intensities per protein, grouped into protein molecular weight bins. The measured protein intensities correlated with the used acrylamide concentration over the whole mass range with less prominent differences in the very high and very low molecular weight of proteins. We observed better immobilization of proteins at higher acrylamide concentrations independent of molecular mass, which is probably a consequence of the smaller pore size of the polyacrylamide matrix. Interestingly, if compared to the binned number of human proteins in Uniprot (20 264 entries, 04/2014), the number of proteins in the database correlated well with the intensities of the identified proteins per mass range at all acrylamide concentrations examined (Fig.1B, secondary axis), possibly because the copy number of a large proportion of proteins are within a limited dynamic range [7]. The recovery of the peptides after in-gel digest has always been a concern as it depends on their size/hydrophobicity and the pore size of the gel matrix. To address if the acrylamide concentration in GASP introduces a systematic bias towards the size and amount of identified peptides, we plotted summed peptides intensities in mass bins (Fig.1C). Our results show that the summed ion intensity of the recovered peptides is higher with increased acrylamide concentration. This is somewhat surprising as a better recovery with a greater pore size of the gel matrix (lower acrylamide concentration) would be expected. We also noticed that the size of gel pieces after shredding is smaller when higher acrylamide concentrations are used. This effect could contribute to the observed results. However, with a smaller pore size, more total protein is immobilized (see above and Fig.1B). Therefore, peptide recovery not only depends on the pore size, but also on the protein amount immobilized. We plotted the total ion intensities of immobilized proteins versus recovered peptides and calculated the peptide/protein intensity ratio at different acrylamide concentrations (Fig.1D), revealing that 18–20% acrylamide concentration is optimal for protein immobilization and at the same time maximal peptide recovery. Our results suggest that the better retention of proteins at higher acrylamide concentrations outweighs a decreased peptide recovery at higher acrylamide concentrations.

As we positioned GASP as an alternative to in-solution and FASP methods, we compared the three methods with 100 μg of a total cell extract prepared with 1% NP-40 (in-solution) or 4% SDS (FASP, GASP). The mechanistically most unrestricted approach regarding protein/peptide size and substrate retention in a lysate should be the in-solution digest as no filtering/immobilization steps are involved. We therefore compared the mass distributions of proteins identified from GASP and FASP prepared samples with in-solution digestion, and found them to be comparable (Supporting Information Fig. 1). Therefore, none of the methods introduces a systematic bias towards protein size. We also compared GASP with FASP and in-solution methods for overall protein identification and spectrum usage and observed that all methods yielded comparable results (Supporting Information Table 1) regarding number of identified peptides/proteins and subcellular localization of identified proteins.

FASP requires a centrifugation of peptides through a molecular weight cut-off filter, while in GASP peptides have to be extracted from the gel matrix. We tested whether FASP and GASP introduce a bias towards molecular weight of eluted peptides (Supporting Information Fig. 2) or their hydrophobicity (Supporting Information Fig. 3) as compared to in-solution protocols. All three methods exhibit the same profile when plotting summed peptide intensities over mass bins. However, we noted that the observed peptide intensities using FASP and GASP are about five times higher compared with in-solution digest despite using the same amount of starting material, possibly due to less effective digest in solution. Consistent with this, FASP and GASP produced less missed cleavage sites than in-solution digest, pointing towards a better solubilization and increased accessibility of cleavage sites to trypsin in those samples. The mean protein coverage was increased in the GASP processed sample (24.9%) over in-solution (24.2%) and FASP (22.7%) processing. In summary, these results suggest that GASP and FASP produce samples of similar abundance and quality from total cell extracts. We expected an increased number of membrane protein IDs with FASP or GASP due to better solubilization over the in-solution digest, but could not verify this in our data.

One particular feature of GASP is the embedment of cysteine alkylation as part of the process of polyacrylamide matrix formation. To assess not only the completeness of conversion of cysteine to PAM-cys, but also to evaluate the formation of other adducts in presence of monomeric acrylamide or ammonium persulfate, we analyzed the data with ProteinPilot (PP, V4.0, ABSciex) (Supporting Information Table 2), a software that uses the Paragon algorithm [19] and is capable to detect unexpected modifications in addition to systematic mass shifts on peptides in an unbiased way. All samples that have been reduced with DTT and alkylated with iodoacetamide achieved high efficiency (99.69% for in-solution digest and 100% for FASP). However, we observed undesired side reactions, such as carbamylation and carbamidomethylation of N-termini, or carbamidomethylation of lysine and some other amino acid residues, interfering with their identification using traditional search algorithms such as MASCOT and SEQUEST with standard search parameters. The modification of cysteine with monomeric acrylamide during the GASP protocol is almost complete (99.83%). Interestingly, much less undesired side reactions were detected in the presence of chaotropes (6 M urea and 2 M thiourea). We also noticed a markedly higher absolute number of cysteine-containing [20] peptides identified in the GASP sample as compared to the other protocols.

To evaluate the scalability of the method, we conducted GASP with different amounts of protein extract ranging from 100 ng to 100 μg in the same matrix volume of 100 μL (Fig.2A). We analyzed relative amounts of the samples representing 100 ng on column for all conditions. When starting with 100 ng of extract, we observed a decrease in protein identifications of approximately 40% as compared to analyzing 10% of a 1 μg GASP run, indicating minimal sample loss when performing GASP on amounts smaller than 1 μg of cell extract. We also performed GASP using counted intact cells as starting material ranging from 50 to 10 000 cells. Cells were lysed using NP-40/DTT buffer and copolymerized and further processed without clearing. We were able to identify 316 proteins from as little as 50 cells (Fig.2B). The number of identifications plateaus at 5000 cells partly due to undersampling of the MS instrumentation at the LC-MS settings used. GASP can be scaled up easily as only the volume of the gel matrix needs to be adjusted. We demonstrated the scalability of GASP with a 1000 μg sample, prepared in a gel matrix of 1000 μL (Fig.2B). The identification of a similar number of proteins when injecting 0.01% of this sample, which equals calculated 100 ng on column, demonstrates that GASP can be performed with higher amounts of cell extract at the same efficiency as with smaller protein amounts—a result which may be difficult to reproduce with in-solution or filter-assisted methods.

**Figure 2:**
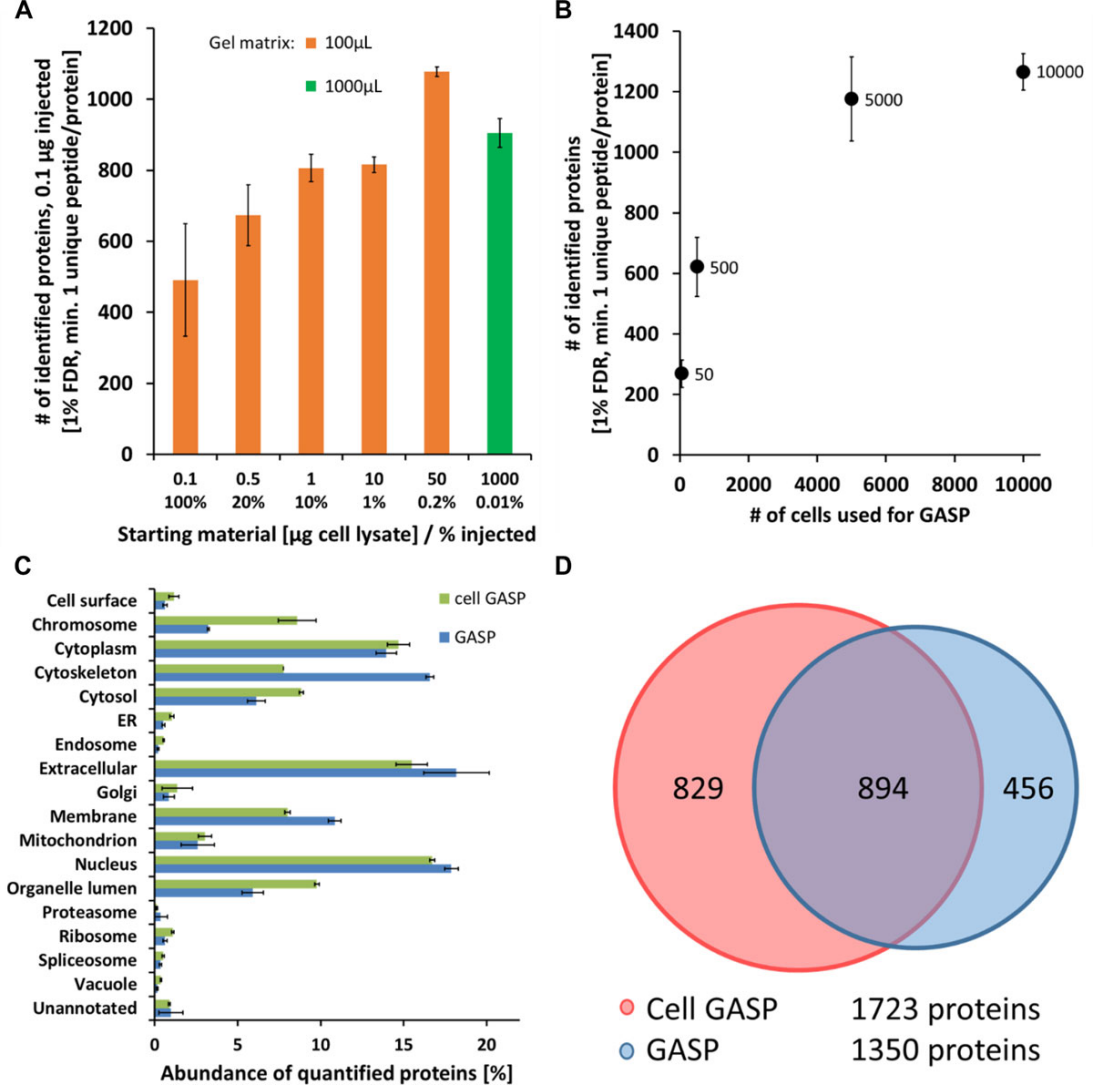
Sensitivity and reproducibility of GASP with different sample amounts and types. (A) GASP was applied to cell extracts ranging from 100 ng to 50 μg in a gel volume of 100 μL and 1 mg in a gel volume of 1 mL in triplicates. Percentages of the generated samples to match 100 ng on column were analyzed with LC-MS. The number of identified proteins shows improving reproducibility with higher amounts of starting material (>1μg). (B) Scepter (Millipore) counted cells were lysed and further processed with GASP. We observed good sensitivity by identification of up to 316 proteins from only 50 cells starting material. (C) Comparison between GASP applied to crude/uncleared cell lysate and intact cells (cell GASP). While subcellular profile of identified proteins is similar (Supporting Information Fig. 4), the relative abundance of chromosome binding and organelle lumen derived proteins as well as the total number of identified proteins (D) is increased by cell GASP-based sample processing.

We propose that GASP can be used on a large variety of samples, such as IPs, homogenized tissues, or small intact organs (i.e., Malpighian tubules from *Drosphila*, unpublished data). To demonstrate the versatility of GASP, we copolymerized PBS suspended, intact cells directly (cell GASP). The sample was then processed in the same way as a cell extract (see also Supporting Information Methods). We achieved an even higher number of protein IDs than in the protein extracts from the same cell number (equivalent of 5000 cells on column, Fig.2D). However, we observed that even though the subcellular profile of the sample is unchanged (Supporting Information Fig. 3), the abundance of proteins derived from organelle lumen and chromosomes is increased in the sample when intact cells are processed with GASP (Fig.2C).

In summary, GASP combines the advantages of in-solution and filter-based methods with the simple, robust, and well-established protocols for in-gel sample preparation without the need of alkylation, precipitation, filtering, or electrophoresis steps. Gel-assisted methods—pioneered originally by Lu and Zhu [4]—have been used previously predominantly on membrane preparations using low acrylamide concentrations. We demonstrate that a smaller pore size at 20% acrylamide minimizes sample loss and enables a highly sensitive detection of proteins in total cell extracts from only 50 cells. Furthermore, GASP can be applied to intact cells or cell extracts between 1 and 1000 μg without loss in efficiency or sensitivity. The GASP protocol can eliminate alkylation and even cell lysis steps from sample preparation for proteomics and is a simple way for producing high-quality samples for MS for nonspecialists.

In comparison to FASP, we did not observe significant differences in identified protein numbers and abundance, but found GASP less prone to sample loss (filter failure, SDS carry over) in our hands and less time-consuming on smaller sample series. Clear advantages are the scalability and the omission of reduction/alkylation steps in GASP, as well as its application to intact cells.

## Abbreviations

FASP: filter-aided sample preparation
GASP: gel-aided sample preparation
PAM-cys: cys-S-β-propionamide
