# Supplementary material for: Gel-aided sample preparation (GASP)—A simplified method for gel-assisted proteomic sample generation from protein extracts and intact cells
Source: Proteomics. 2015 Feb 5;15(7):1224–9. doi: 10.1002/pmic.201400436 (PMC4409837; doi:10.1002/pmic.201400436)
Supplement: Supplementary file 1 [file pmic0015-1224-sd1.docx]

**Gel-aided sample preparation (GASP) – a simplified method for gel-assisted proteomic sample generation from protein extracts and intact cells**

^1,2^Roman Fischer and ^1^Benedikt M. Kessler

^1^TDI Mass Spectrometry Laboratory, Target Discovery Institute, Nuffield Department of Medicine, University of Oxford, Roosevelt Drive, Oxford OX3 7FZ, UK

^2^Corresponding author

**Supplementary figures and tables:**

**Supplementary Fig. 1 Comparison of proteins identified after GASP with FASP and regular in-solution digest**

**Supplementary Fig. 2 Comparison of peptides identified after GASP with FASP and regular in-solution digest**

**Supplementary Fig. 3 Hydrophobicity of peptides identified using GASP, FASP or in-solution digest**

**Supplementary Fig. 4 Subcellular annotation of proteins identified in GASP and cell GASP**

**Supplementary Tab. 1 Comparison of data characteristics after LC-MS/MS analysis of cell extracts prepared with GASP, FASP and in-solution digest**

**Supplementary Tab. 2 Side reactions and their frequencies induced by differential sample preparation methods**

**Supplementary methods**

**Supplementary Fig. 1 Comparison of proteins identified after GASP with FASP and regular in-solution digest.** (**a**) 100 μg of cell extract prepared from 4% SDS lysates (FASP and GASP) or 1% NP40 lysate (in-solution) were prepared and 1% of the digested sample analysed with LC-MS. Signal intensities of identified proteins were extracted using Progenesis LCMS (V4.1.4832.42146) summed in mass bins. While the number of identified proteins is comparable between the methods, FASP and GASP samples were generally more abundant. The profile of identified proteins in FASP and GASP matches the distribution of in-solution digest – a method that is less prone to introducing bias due to the lack of protein immobilisation. (**b**) Identified proteins and their overlap using GASP, FASP and in-solution digest. LC-MS parameters were optimized to detect ion count differences consequently resulting in under-sampling in all samples, hence the similar number of identifications. (**c**) Principal Component Analysis (PCA) using the abundance of identified proteins in the three compared samples (duplicate analysis). 701 out of 975 proteins were detected with ANOVA p≤0.05 and used as component loadings to demonstrate the difference between the samples prepared with in-solution digest, FASP and GASP on the protein level. (**d**) Label-free quantitation of identified proteins shows that GASP and FASP provide a more effective environment for tryptic digestion than in-solution to prepare bottom-up samples from total cell lysates. Most proteins are detected along the diagonal, demonstrating similar sample properties.


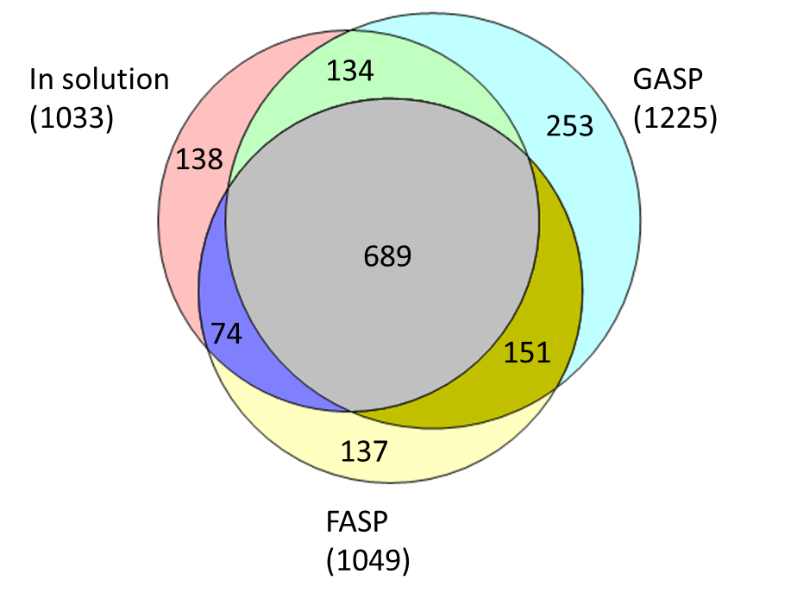

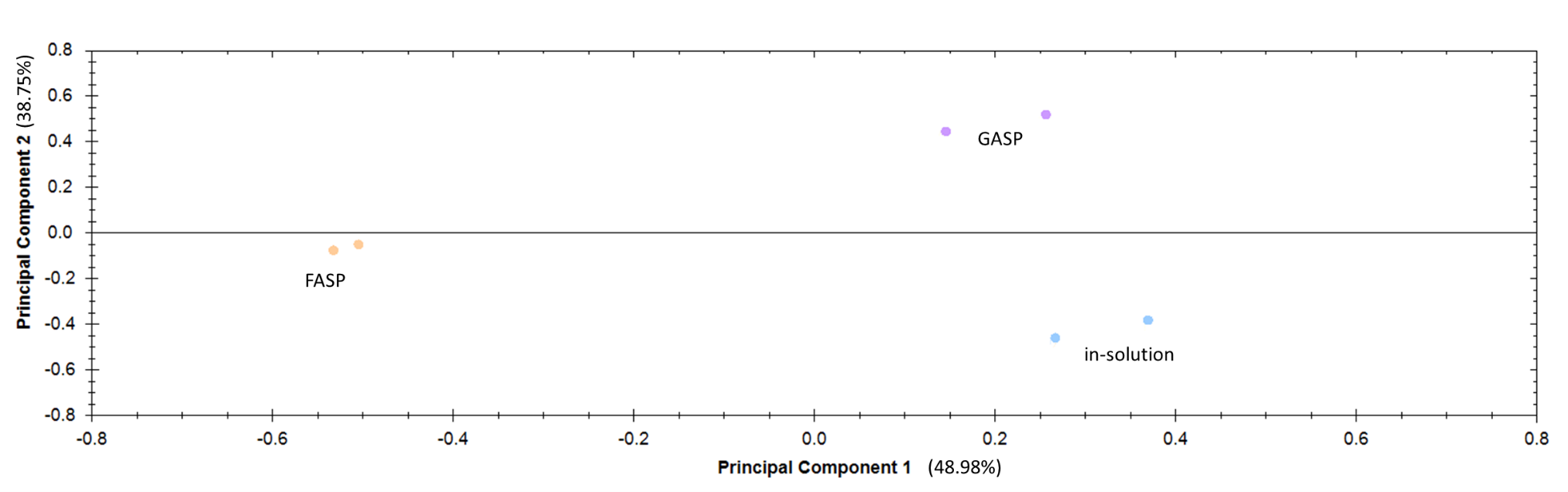


**c**

**b**

**a**


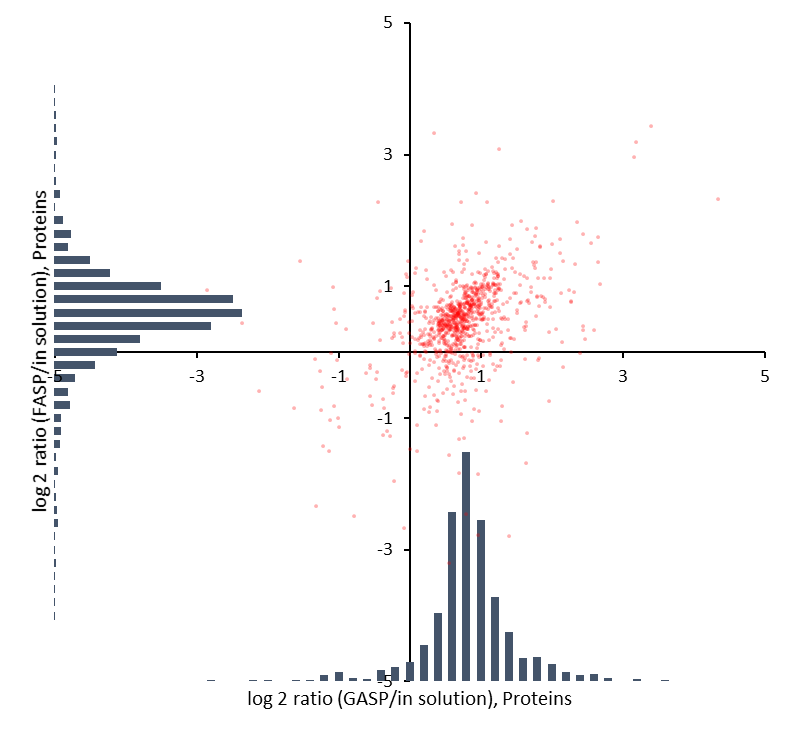


**d**

**Supplementary Fig. 2 Comparison of peptides identified after GASP with FASP and regular in-solution digest.** (**a**) The distribution of identified peptides shows no systematic bias towards peptide mass comparing FASP and GASP with in-solution digest. (**b**) Number of identified peptide sequences and their overlap using GASP, FASP and in-solution digest. GASP shows the biggest overlap between the three methods. (**c**) Principal Component Analysis using the abundance of identified peptides in the three compared samples (duplicate analysis). 5721 out of 7926 peptides were detected with ANOVA p≤0.05 and used as component loadings to demonstrate the difference between the samples prepared with in-solution digest, FASP and GASP on the peptide level. (**d**) Label-free quantitation of identified peptides shows that GASP and FASP shows a similar result than the quantitation on the protein level. GASP and FASP yield in increased abundance of peptides. This histogram demonstrates a similar abundance in the FASP and GASP processed samples for the majority of detected peptides. However it has to be taken into account that GASP and FASP treated cells have been lysed with SDS while NP40 was used on the in-solution digested cell extract, yielding in differential protein and peptide abundance.
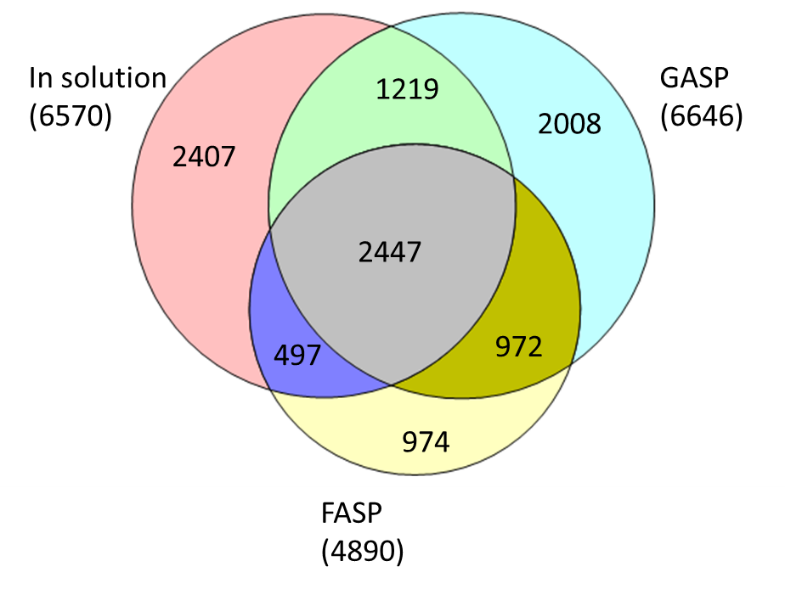

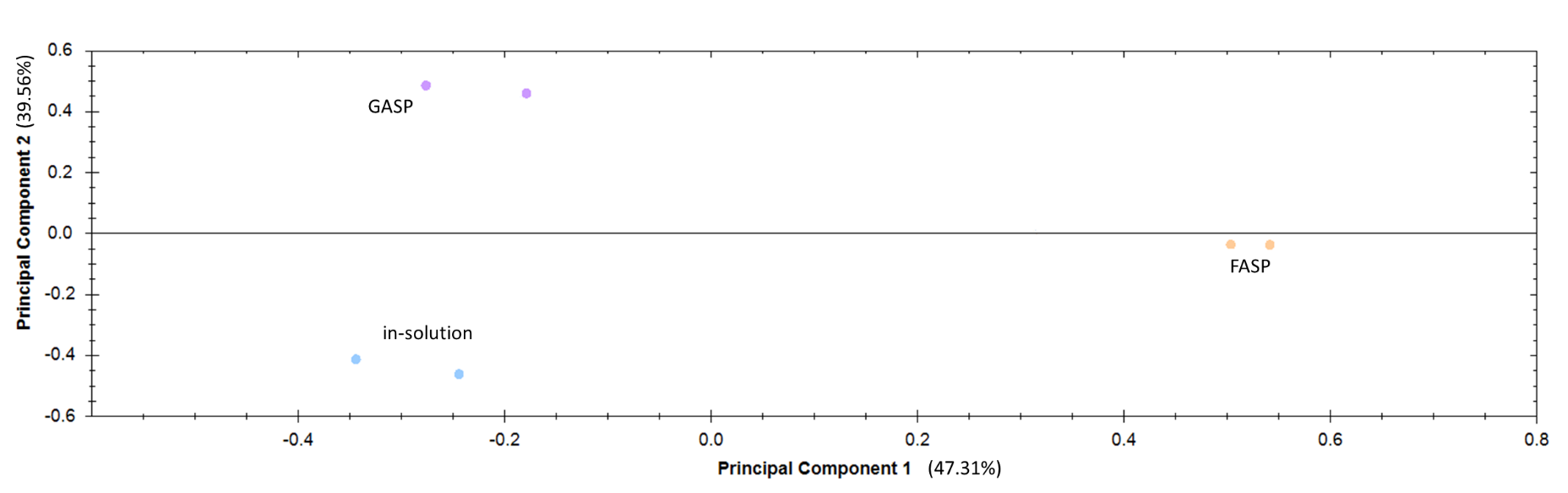


**a**

**b**

**c**


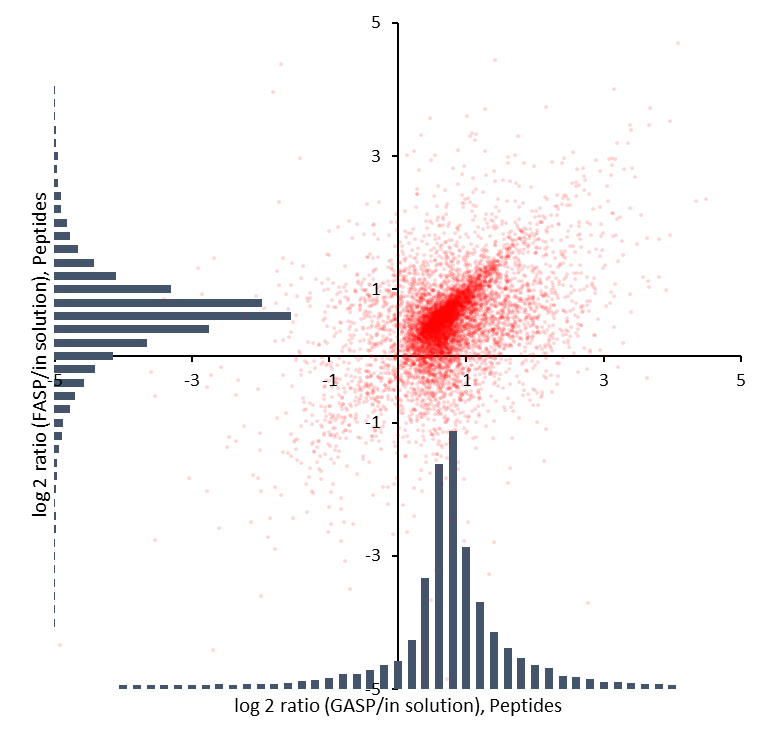


**d**

**Supplementary Fig. 3 Hydrophobicity of peptides identified using GASP, FASP or in-solution digest.** The hydrophilic index of identified peptides (Hopp and Woods[[1](#_ENREF_1)]). While GASP and FASP perform equally well with hydrophilic peptides (positive values), GASP performs better with hydrophobic peptides (negative values), when compared to the principally unbiased in-solution digest.

**Supplementary Fig. 4 Subcellular annotation of proteins identified in GASP and cell GASP.** The distribution of subcellular annotations is similar when cell extracts or intact cells are prepared with GASP. However the abundance of chromosome binding and organelle lumen derived proteins appears higher when intact cells are copolymerized with acrylamide (**Fig. 2c**).

**Supplementary Tab. 1 Comparison of data characteristics after LC-MS/MS analysis of cell extracts prepared with GASP, FASP and in-solution digest.** LC-MS/MS setting and injection amount were chosen to procure a maximum of identifications in the reference sample (in-solution digest). Therefore the results reflect sample quality. Sample abundance could be assessed visualising the results as shown in Supplementary Figures 1 and 2 (by signal intensity), instead of improving peptide separation (i.e. longer LC gradient) or adjusting injection amount. Data were analysed with Mascot (V2.3) or ProteinPilot (PP, V4.0). Besides the small differences in total identified peptides (peptide spectrum matches, PSMs) and proteins, the proteolysis appears to be more effective in GASP and FASP as reflected by the higher percentage of missed cleavage sites observed in in-solution digest. Remarkably, more cysteine containing peptides could be identified in GASP processed samples. The number of side reactions due to incubation with acrylamide is lower than the number of side reactions induced by the incubation with acrylamide (for details see Supplementary Tab. 2).

**Supplementary Tab. 2 Side reactions and their frequencies induced by differential sample preparation methods.** Modified peptides were identified by ProteinPilot (ABSciex, Version 4.0) due to its ability to conduct unrestricted PTM analysis [[2](#_ENREF_2)]. In-solution digests suffer from carbamylation reactions due to the presence of prolonged presence of 1M urea during the digest. However, cysteine alkylation derived side reactions are at a minimum as DTT and Iodoacetamide are removed prior to proteolysis by protein precipitation. FASP processed samples show in our hands an increased number of side reactions derived from the presence of DTT and Iodoacetamide. A possible explanation could be the long centrifugation times in addition to the incubation to facilitate complete reduction and alkylation. GASP exhibits the lowest number of undesired side reaction with propionamide modifications on lysine and N-termini in presence of chaotropic agents (6M Urea and 2M Thiourea). We hypothesize that a prolonged incubation of the sample in monomeric acrylamide prior to polymerisation may increase this percentage due to the high stoichiometric excess of acrylamide. Prolonged incubation should be avoided.

**Supplementary methods**

**Cell Extract preparation**

For the evaluation of the ideal acrylamide concentration, HEK 293T cell lysates of equal cell numbers were prepared by NP40 lysis (1% NP40, 100mM Tris (pH7), Mini Complete (Roche)) for in solution digest or SDS lysis (4% SDS, 100mM DTT, 6M Urea, 1.5M Thiourea) for GASP and FASP protocols. After clearing by centrifugation, protein concentration was measured (NP40 lysate) and 100ug adjusted to a volume of 100ul prior to GASP.

For the evaluation of GASP sensitivity we used cleared Jurkat cell extracts after lysis with NP40 buffer. Protein concentration was measured with BCA assay (Thermo, Pierce). DTT and SDS were added to a final concentrations of 50mM and 2% respectively. Protein amounts ranging 100ng to 50ug were adjusted to a volume of 50µl with lysis buffer before the GASP procedure. The upscaling experiment was conducted with 1mg of protein extract and an adjusted volume of 500µl. The experiment was conducted in triplicates.

The cell based sensitivity evaluation of GASP was conducted using T24 bladder cancer cells, grown in DMEM medium supplemented with 10% fetal calf serum (FCS) and 1% penicillin/streptomycin and 1% glutamine. Cells were counted with a Scepter automated cell counter (Millipore) in PBS. 50 to 10,000 cells were diluted in PBS to a volume of 12.5µl and adding 12.5µl DTT/NP40 lysis buffer (as above, supplemented with 50mM DTT), resulting in a total volume of 25µl prior to GASP. The experiment was conducted in triplicates.

For cell GASP, 40,000 intact T24 cells were adjusted to a total volume of 50µl in PBS. For the comparison with a sample prepared from 5,000 T24 lysed cells, 1/8 of the sample was analysed. The experiment was conducted in duplicates.

**Gel-aided sample preparation (GASP)**

Protein extracts were left at room temperature after adding DTT for 20 minutes for disulphide bond reduction. An equal volume Protogel (40% w/v, 37.5:1 Acrylamide:Bisacrylamide solution, National Diagnostics) was added, mixed by gentle pipetting and left at room temperature for 20 minutes. 5µl of Tetramethylethylenediamine (TEMED) and 5µl of 10% Ammonium Persulfate (APS) were added. The sample was left to polymerize for 5-10 minutes until the gel was solid and the resulting gel plug was transferred to an acetate membrane SpinX centrifugation filter in which the filter membrane had been removed previously by desolvation in organic solvent (.i.e. acetone). The remaining filter membrane support was used to cut the gel pieces into small cubes by pulse centrifugation. The gel pieces were then fixed by adding 1ml of methanol/acetic acid/water (50/40/10) for 10 minutes in an overhead mixer. After pulse centrifugation the supernatant was discarded, 500µl of 6M Urea was added and the gel pieces were washed for 10 minutes in a rotator. To dehydrate the gel pieces and to alleviate the removal of the supernatant 1ml of acetonitrile was added. In most cases a single Urea/ACN wash cycle will suffice to remove SDS and other contaminants, however if higher concentrations are used we recommend to repeat the Urea/ACN wash. The dehydration step was followed by rehydration in 500µl 50mM Triethylammonium bicarbonate (TEAB) and rotation for 10 minutes. The gel pieces were dehydrated by addition of 1ml Acetonitrile. After discarding the supernatant the gel pieces were dried further by adding 500µl Acetonitrile until the gel pieces agglomerate at the bottom of the tube. Trypsin solution (1/50 enzyme substrate ratio when protein concentration was known and 20ng/ml when protein concentration was unknown) of the same volume as the original gel plug was added to the dry gel pieces for proteolysis at 37°C over night.

Peptide extraction was facilitated by first adding 1 volume of acetonitrile. The supernatant was transferred into a new tube and the gel pieces were rehydrated in 5% formic acid, followed by dehydration with 1 volume of acetonitrile. Supernatants were combined after further dehydration in 1 volume of Acetonitrile. The samples were dried in a vacuum concentrator and resuspended in 0.1% Trifluoroacetic acid in 1% Acetonitrile for LC-MS analysis.

**Gel-aided sample preparation of intact cells (cell GASP)**

50µl of Acrylamide solution was added to 50µl of (40,000) intact and PBS-suspended cells, followed by polymerisation with 5µl TEMED and 5µl APS. The Gel plug was shredded as described before and fixed in 1ml of fixing solution. The urea wash was followed by incubation in 10mM DTT and 50mM Iodoacetamide prior to dehydration and buffer exchange to 50mM TEAB and tryptic digest as described above.

**General notes:**

- The SpinX gel cutting units can be reused after rinsing.
- The presence of Urea and Thiourea in the lysis buffer reduces the reaction of N-termini and lysine with monomeric acrylamide and helps solubilisation of proteins (recommendation 6M Urea and 1.5M Thiourea).
- Supernatants should only be removed after acetonitrile was added, as the partially dehydrated gel pieces will agglomerate. This notably alleviates the liquid handling and avoids the loss of gel pieces.
- Supernatants should be removed by using gel loader tips (they can be stacked on a 1ml pipette tip) to avoid the loss of gel pieces.
- Samples can be split easily at any step of the procedure, i.e. if different proteolytic enzymes are used.
- Gel pieces can be used as micro reactors to facilitate highly effective (bio)-chemical reactions such as protein labelling.
- Additional desalting is usually not necessary (TEAB can be replaced with Ammonium bicarbonate)
- GASP will yield peptides with propionamide on lysine and N-termini. These modifications should be considered as variable in database searches.

**Mass Spectrometric Analysis**

Dry samples were reconstituted in 0.1% TFA in 1% Acetonitrile and desalted on line on a trap column (Details below). The ideal Acrylamide concentration was determined on an Orbitrap Velos mass spectrometer (Thermo) coupled to a nAquity UPLC system (Waters). The setup was described before [[3](#_ENREF_3)].

Briefly, samples were desalted online (Symmetry C18 column (180 µm x 20mm, 5 µm particle, Waters) for 5 minutes at a flow rate of 5µl/min, followed by separation on a BEH C18 column (75 μm × 250 mm, 1.7 μm particle, Waters) over 60 minutes using a gradient of 2%-40% Acetonitrile in 0.1% Formic acid at 250nl/min. Survey scans were acquired in the Orbitrap at a resolution of 60.000 @ 400m/z and the 20 most abundant precursors were selected for CID fragmentation.

A Q Exactive mass spectrometer coupled with a Dionex Ultimate 3000 UPLC was used to explore the sensitivity of GASP with cell extracts down to 100ng or 50 cells starting material. Samples were desalted online (PepMAP C18, 300µm x5mm, 5 µm particle, Thermo) for 1 minute at a flow rate of 20 µl/min and separated on a nEASY column (PepMAP C18, 75 µm x 500mm, 2 µm particle, Thermo) over 60 Minutes using a gradient of 2%-35% Acetonitrile in 5% DMSO/0.1% Formic acid at 250nl/min[[4](#_ENREF_4)]. Survey scans were acquired at a resolution of 70,000 @ 200m/z and the 15 most abundant precursors were selected for HCD fragmentation.

The selected conditions for both LC-MS/MS platforms result in under sampling throughout the gradient and were chosen to focus on low sample abundance and method sensitivity rather than identification numbers.

**Data analysis**

Were necessary peak list files were generated with MSConvert (Proteowizard V3.0.5211) using the 200 most abundant peaks/spectrum. Depending on the addressed question we used Mascot (V2.3, Matrix Science), PEAKS (V7, Bioinformatics Solutions), ProteinPilot (V4.0, ABSciex) and ProteomeDiscoverer (V1.4, Thermo), all at a false discovery rate of 1%, mass deviation of 10ppm for MS1 and 0.5 Da (Orbitrap Velos)/0.06 Da (Q Exactive) for MS2 spectra.

The acrylamide concentration (Orbitrap Velos) was evaluated with results from Mascot. GASP, FASP and in solution (Orbitrap Velos) were compared with data analysed with Mascot and ProteinPilot, while the sensitivity of GASP was evaluated with Mascot and PEAKS. Ion intensities were extracted with Progenesis LCMS (V4.1.4832.42146) and subcellular annotations and their quantitation were analysed with ProteomeDiscoverer. The mass distribution of the human proteome was calculated with Protein Digestion Simulator by Matthew Monroe (http://omics.pnl.gov/software/protein-digestion-simulator).

Different search engines were used to best address the specific questions in each experiment. The algorithms were chosen with the following reasoning:

Figure 1: optimal acrylamide concentration – introduced PTMs, digest efficiency and sensitivity were less relevant in the experimental context, so we chose Mascot as fast search algorithm.

Figure 2: Sensitivity evaluation – we used PEAKS for the evaluation of sensitivity as its PTM search can detect all modifications present in Unimod and it has superior FDR calculations and superior sensitivity compared to other algorithms.

Suppl. Table 1 and 2: Method comparison (GASP vs. FASP vs. in-solution) – We used Mascot for basic searches (Protein IDs) because of speed and ease of use. To evaluate digest efficiency and modifications we used ProteinPilot as its unique search algorithm allows to detect all modifications listed in Unimod and also unknown mass shifts. A statistical template allows easy access to PTM and digest statistics.

**Filter-aided sample preparation (FASP)**

FASP samples were prepared as described by Wisniewski et al. [[5](#_ENREF_5)] using a Spin-X UF500 10k MWCO filter (Corning) after lysis in SDS/DTT buffer (see online methods). Briefly 100µg of lysate was adjusted to a volume of 100µl with lysis buffer before mixing with 200µl of 8M Urea and centrifugation followed by Urea washes as described, cysteine alkylation with Iodoacetamide and tryptic digest. Peptides were desalted with SPE Plus cartridges (Waters).

**In-solution digest**

Cells were lysed in NP40 buffer. 100µg cell extract was reduced and alkylated before precipitation with Chloroform/Methanol [[6](#_ENREF_6)]. The protein pellet was resuspended in 100 µl 6M urea for solubilisation. The sample was diluted to 1M Urea in 100mM TEAB for tryptic digest. Peptides were desalted with SPE Plus cartridges (Waters).

[1] Hopp, T. P., Woods, K. R., Prediction of protein antigenic determinants from amino acid sequences. *Proceedings of the National Academy of Sciences of the United States of America* 1981, *78*, 3824-3828.

[2] Shilov, I. V., Seymour, S. L., Patel, A. A., Loboda, A.*, et al.*, The Paragon Algorithm, a next generation search engine that uses sequence temperature values and feature probabilities to identify peptides from tandem mass spectra. *Molecular & cellular proteomics : MCP* 2007, *6*, 1638-1655.

[3] Fischer, R., Trudgian, D. C., Wright, C., Thomas, G.*, et al.*, Discovery of candidate serum proteomic and metabolomic biomarkers in ankylosing spondylitis. *Molecular & cellular proteomics : MCP* 2012, *11*, M111 013904.

[4] Hahne, H., Pachl, F., Ruprecht, B., Maier, S. K.*, et al.*, DMSO enhances electrospray response, boosting sensitivity of proteomic experiments. *Nature methods* 2013, *10*, 989-991.

[5] Wisniewski, J. R., Zougman, A., Nagaraj, N., Mann, M., Universal sample preparation method for proteome analysis. *Nature methods* 2009, *6*, 359-362.

[6] Wessel, D., Flugge, U. I., A method for the quantitative recovery of protein in dilute solution in the presence of detergents and lipids. *Analytical biochemistry* 1984, *138*, 141-143.
